# Correction: Exploring the potential of nest archives for establishing long-term trends in local populations of an Arctic-nesting colonial sea duck

**DOI:** 10.1371/journal.pone.0344590

**Published:** 2026-03-09

**Authors:** Inmaculada Álvarez-Manzaneda, Kathleen M. Rühland, Marlo Campbell, Matthew P. Duda, Mark L. Mallory, Nik Clyde, H. Grant Gilchrist, Kathryn E. Hargan, John P. Smol

The last two sentences of Fig 9 caption should be ommited. Please see the complete, correct Fig 9 caption here.

In the Radioisotopic dating (210Pb and 14C) subsection of the Materials and methods, there is an error in the eighth and ninth sentences of the first paragraph. The correct sentences are: The chronologies for the nest profiles were based on estimates of unsupported 210Pb activities and the constant rate of supply (CRS) model [35], using the ScienTissiME dating software (http://www.scientissime.net/software). The CRS model is widely used for dating recent sediment profiles [34,35] and assumes a constant supply of unsupported 210Pb deposited from the atmosphere to the surface of vertically aggrading profiles, while allowing for variations in accumulation rates [35].

In the Radioisotopic dating (210Pb and 14C) subsection of the Results, there is an error in the first sentence of the second paragraph. The correct sentences are: Establishing chronologies for the nest profiles DS-E5-N1 and DS-E3-N1 entailed combining 210Pb and 14C dating results, as the bottom intervals of these nests lie beyond the maximum range possible for obtaining reliable dates using 210Pb (~150 years) methods but were within the upper range of 14C methods.

The Data Availability statement for this article is incorrect. The correct statement is: All data, figures and scripts used in the manuscript are uploaded to Zenodo. The DOI is: https://doi.org/10.5281/zenodo.15526799.

**Fig 9:**
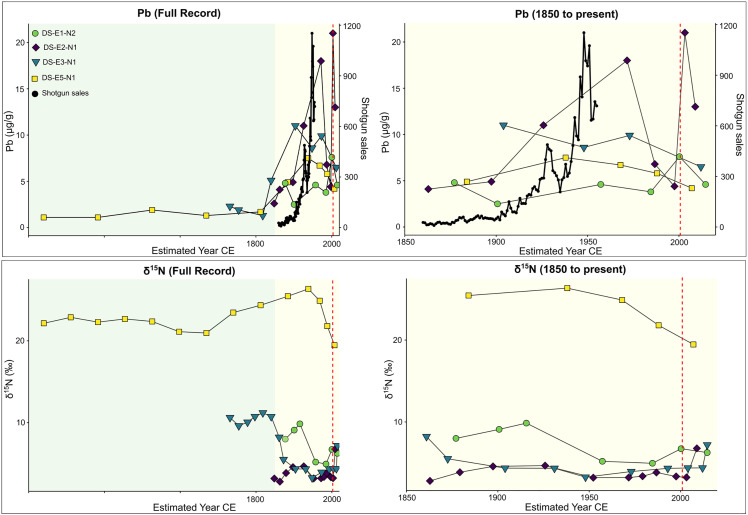
Total stable Pb concentrations (µg/g) plotted against 210Pb dates for the four Digges Sound eider nest profiles studied, compared to the recorded number of shotguns traded or sold in West Greenland between 1860 and 1955 (top panel; [62]). Coloured areas distinguish the pre-industrial (green) and post-industrial (yellow) eras. The vertical red dotted line indicates the start of regulated hunting in Greenland. The figure on the right presents a zoomed-in view highlighting trends during the post-industrial era. Sedimentary δ15N (‰) plotted against 210Pb dates for the four eider nest profiles are shown in the bottom panel.
