# Supplementary figures and images for: Exploring the potential of nest archives for establishing long-term trends in local populations of an Arctic-nesting colonial sea duck
Source: PLoS One. 2025 Oct 10;20(10):e0332605. doi: 10.1371/journal.pone.0332605 (PMC12513636; doi:10.1371/journal.pone.0332605)

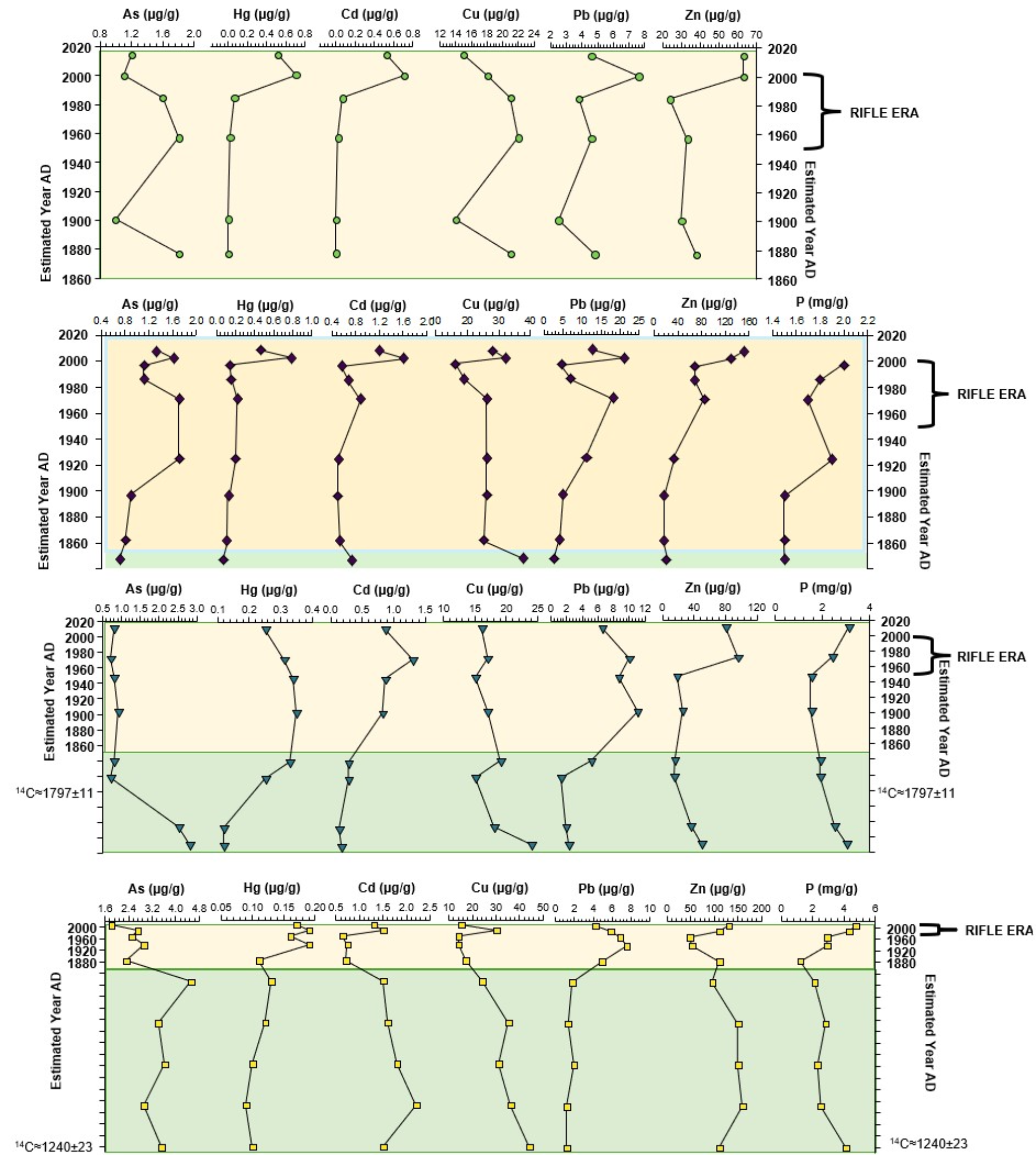

Supplement: S1 Fig — Elemental concentrations are either presented in μg g−1 or mg g−1 dry weight as stated. (TIF) [file pone.0332605.s001.tif]
