# Supplementary material for: Exploring the potential of nest archives for establishing long-term trends in local populations of an Arctic-nesting colonial sea duck
Source: PLoS One. 2025 Oct 10;20(10):e0332605. doi: 10.1371/journal.pone.0332605 (PMC12513636; doi:10.1371/journal.pone.0332605)
Supplement: S1 Table — (PDF) [file pone.0332605.s002.pdf]

Lalonde AMS Lab ID	Study ID	Treatment	14C yr BP ±	F14C ±	Cal BP
UOC-18699	DS-E3-N1 11-12cm	AAA	142 ± 11	0.9825 ± 0.0013	273 - 250 (14.5%)<br>230 - 208 (12.6%)<br>200 - 186 (4.5%)<br>153 - 135 (10.5%)<br>118 - 61 (32.0%)<br>43 - 8 (21.3%)
UOC-18700	DS-E5-N1 13-14 cm	AAA	819 ± 11	0.9031 ± 0.0013	733 - 688 (95.4%)
