# Supplementary material for: Exploring the potential of nest archives for establishing long-term trends in local populations of an Arctic-nesting colonial sea duck
Source: PLoS One. 2025 Oct 10;20(10):e0332605. doi: 10.1371/journal.pone.0332605 (PMC12513636; doi:10.1371/journal.pone.0332605)
Supplement: S2 Table — (PDF) [file pone.0332605.s003.pdf]

**Species**	**Authority**
Pinnularia borealis	Ehrenberg, 1843
Pinnularia intermedia	(Lagerstedt) Cleve, 1895
Pinnularia divergentissima	(Grunow) Cleve, 1895
Luticola mutica	(Kützing) D.G. Mann, 1990
Hantzschia amphioxys	(Ehrenberg) Grunow, 1880
Cosmioneis pusilla	(W.Smith) D.G.Mann & A.J.Stickle, 1990
Mayamaea atomus	(Kützing) Lange-Bertalot, 1997
Humidophila perpusilla	(Grunow) R.L.Lowe, Kociolek, J.R.Johansen, Van de Vijver, Lange-Bertalot & Kopalová, 2014
Pinnularia sinistra	Krammer, 1992
Stauroneis gracilis	Ehrenberg, 1843
Pinnularia viridiformis	Krammer, 1992
Navicula sublinearis	Grunow, 1885
Nitzschia inconspicua	Grunow, 1862
Pinnularia dubitabilis	Hustedt, 1949
Navicula phyllepta	Kützing, 1844
Pinnularia minutiformis	Krammer, 2000
Cocconeis costata	W. Gregory, 1885
Nitzschia linearis	W..Smith, 1853
