# Supplementary material for: Exploring the potential of nest archives for establishing long-term trends in local populations of an Arctic-nesting colonial sea duck
Source: PLoS One. 2025 Oct 10;20(10):e0332605. doi: 10.1371/journal.pone.0332605 (PMC12513636; doi:10.1371/journal.pone.0332605)
Supplement: S1 File — (DOCX) [file pone.0332605.s004.docx]

**Supplementary Information for**

Exploring the potential of nest archives for establishing long-term trends in local populations of an Arctic-nesting colonial sea duck

Álvarez-Manzaneda, Inmaculada, Rühland, Kathleen M., Campbell, Marlo, Duda, Matthew P., Mallory, Mark L., Clyde, Nik, Gilchrist, Grant, Hargan, Kathryn E., and Smol, John P.

Email: miams@ugr.es

This file includes:

Supplementary text

**Supplementary Methods**

**S1 Text. Stable nitrogen isotopes.** Scale calibration is necessary for every run of the EA-IRMS. We used EDTA #2 (Schimmelmann Research Lab, Indiana University: δ^13^C -40.38, δ^15^N -0.83), caffeine #2 (Schimmelmann Research Lab, Indiana University: δ^13^C -14.79, δ^15^N 20.17), casein (B2155, Elemental Microanalysis: δ^13^C -27.03, δ^15^N 5.97), and glutamic acid (in-house standard, δ^13^C -26.74, δ^15^N -2.77). Acetanilide (δ^13^C -27.14, δ^15^N 0.63) was used to prepare a calibration curve for %C and %N.

**Supplementary Text.**

**S2 Text. Temporal trends in ornithogenic proxies from nest profiles**. In DS-E1-N2, δ^15^N values were higher after its establishment (~6‰), in comparison with DS-E2-N1 and DS-E3-N1, and the trend in δ^15^N shows a decline by ~5‰ around ca. 1925 and an increase around ca. 2000 (Fig. 5b). DS-E2-N1 shows stable δ^15^N values (3-4‰) from ca. 1850s until a notable increase at ca. 2000 (by reaching ~7‰). A small change in δ^15^N values in nest DS-E3-N1 occurs after ca. 2000 with an increase to 7‰. Nest DS-E5-N1 captured δ^15^N values higher than the other nests (mean = ~23.6‰) with a decrease after ca. 1900 until the most recent period.

Regarding the other elements of DS-E3-N1 (Fig. S1c), although most of them remained stable, there were some striking changes in As, Hg and Cu during the pre-industrial period. While As experienced a notable decline from the time when the nest was established until ca. 1850, Hg had the opposite trend, reaching maximum concentrations ca. 1850s, and Cu also decreased although this decline was not that steep. In general, no element showed any notable changes in DS-E5-N1 with the exception of As that experienced a notable decrease after ca. 1820 (Fig. S1d).

Regarding the rest of the elements (Fig. S1), in DS-E1-N2 As and Cu had similar trends, decreasing until ca. 1900 and increasing again after until ca. 1980 when they decreased until the most recent period. In the case of the trends of Hg remained stable until ca. 1980 following by an increase thereafter. For nest DS-E2-N1 (Fig. S1b), the seven selected elements showed relatively low and stable concentrations before ca. 1900. After ca. 1900, As and P concentrations increased, followed by a notable decrease in As and Cu after ca. 1960. In the most recent part of this record (ca. 21^st^ century), it was observed a general increase before decreasing in the last interval. Regarding DS-E3-N1, As and Cu showed a general decline after ca. 1850 to relatively stable concentrations thereafter. In contrast, P notably increased after ca. 1950 until the present (Fig. S1c). In the profile DS-E5-N1 (Fig. S1d), Hg concentrations increased after ca. 1880 and P concentrations increased after ca. 1980 until present.

Increasing anthropogenic pressures on eider populations at the transition to the industrial era was also evident by an increase in several metal(loid)s within the nest profiles. For example, there were general notable increases observed in As, Hg and Cu after ca. 1900 that could also be a consequence of increased human activities during the early to mid-20^th^ century. A clear increase in Hg concentrations was observed in DS-E2-N1, DS-E3-N1 and DS-E5-N1 approximately coincident with the onset of 19^th^ century industrialization in North America (Fig. S1) that may be a consequence of the increase in the use of coal [1]. These elements were found to increase in snow and ice sampled from Greenland, with As concentrations peaking in the late 1890s to the late 1910s [2]. In sediment profiles from Canadian High Arctic lakes, Hg concentrations increased after the 1900s [3]. On the other hand, As concentrations are also high in ponds where eiders and other seabirds are abundant [4,5], indicating that increases in As concentrations may also be a signal of the occupation of the nests by eiders.

**References of the supplementary text**

1. Pérez-Rodríguez M, Silva-Sánchez N, Kylander ME, Bindler R, Mighall TM, Schofield JE, et al. Industrial-era lead and mercury contamination in southern Greenland implicates North American sources. Sci Total Environ. 2018;613–614: 919–930. doi:10.1016/j.scitotenv.2017.09.041

2. Lee K, Han C, Hong S-B, Jun S-J, Han Y, Xiao C, et al. A 300-Year High-Resolution Greenland Ice Record of Large-Scale Atmospheric Pollution by Arsenic in the Northern Hemisphere. Environ Sci Technol. 2019;53: 12999–13008. doi:10.1021/acs.est.9b01805

3. Outridge, Sanei H, Stern, Hamilton, Goodarzi F. Evidence for Control of Mercury Accumulation Rates in Canadian High Arctic Lake Sediments by Variations of Aquatic Primary Productivity. Environ Sci Technol. 2007;41: 5259–5265. doi:10.1021/es070408x

4. Hargan KE, Michelutti N, Coleman K, Grooms C, Blais JM, Kimpe LE, et al. Cliff-nesting seabirds influence production and sediment chemistry of lakes situated above their colony. Sci Total Environ. 2017;576: 85–98. doi:10.1016/j.scitotenv.2016.10.024

5. Duda MP, Hargan KE, Michelutti N, Kimpe LE, Clyde N, Gilchrist HG, et al. Breeding eider ducks strongly influence subarctic coastal pond chemistry. Aquat Sci. 2018;80: 40. doi:10.1007/s00027-018-0591-2
