# Supplementary material for: Exploring the potential of nest archives for establishing long-term trends in local populations of an Arctic-nesting colonial sea duck
Source: PLoS One. 2025 Oct 10;20(10):e0332605. doi: 10.1371/journal.pone.0332605 (PMC12513636; doi:10.1371/journal.pone.0332605)
Supplement: Attachment — Submitted filename: Response to reviewers.docx [file pone.0332605.s006.docx]

We deeply appreciate the comments of the reviewers, and the time dedicated to reviewing our manuscript. We believe that we have been able to effectively respond to all the comments raised in the review process, as detailed below.

Response to Academic Editor:

1. When submitting your revision, we need you to address these additional requirements.

Please ensure that your manuscript meets PLOS ONE's style requirements, including those for file naming.

RESPONSE: The authors have reviewed the manuscript to meet PLOS ONE’s style requirements.

2. Thank you for stating the following financial disclosure: [This research was funded by Environment and Climate Change Canada, the W. Garfield Weston Foundation, a Kenneth M. Molson Foundation grant, the Natural Sciences and Engineering Research Council of Canada (NSERC), Oceans North and the PEW Charitable Trusts, Nunavut General Monitoring Plan, ArcticNet Network Centres of Excellence, and was within the project PAST, financially supported by the European Commission (H2020-MSCA-IF-2019, Grant no. 897535).].

Please state what role the funders took in the study. If the funders had no role, please state: ""The funders had no role in study design, data collection and analysis, decision to publish, or preparation of the manuscript.""

If this statement is not correct you must amend it as needed.

Please include this amended Role of Funder statement in your cover letter; we will change the online submission form on your behalf.

RESPONSE: This amended Role of Funder statement has been included in the cover letter.

3. In the online submission form, you indicated that your data will be submitted to a repository upon acceptance. We strongly recommend all authors deposit their data before acceptance, as the process can be lengthy and hold up publication timelines. Please note that, though access restrictions are acceptable now, your entire minimal dataset will need to be made freely accessible if your manuscript is accepted for publication. This policy applies to all data except where public deposition would breach compliance with the protocol approved by your research ethics board. If you are unable to adhere to our open data policy, please kindly revise your statement to explain your reasoning and we will seek the editor's input on an exemption.

RESPONSE: Following your recommendations, the data used in this research has been deposited in the EU Open Research Repository: Zenodo. (https://doi.org/10.5281/zenodo.15526798)

4. We note that Figure 2 in your submission contain [map/satellite] images which may be copyrighted. All PLOS content is published under the Creative Commons Attribution License (CC BY 4.0), which means that the manuscript, images, and Supporting Information files will be freely available online, and any third party is permitted to access, download, copy, distribute, and use these materials in any way, even commercially, with proper attribution. For these reasons, we cannot publish previously copyrighted maps or satellite images created using proprietary data, such as Google software (Google Maps, Street View, and Earth). For more information, see our copyright guidelines: http://journals.plos.org/plosone/s/licenses-and-copyright.

RESPONSE: Thank you for your comment. With the aim to meet the requirements, OpenStreetMap was used for rendering using QGIS. The base map and data from OpenStreetMap and OpenStreetMap Foundation (CC-BY-SA). © https://www.openstreetmap.org and contributors.
It has been accordingly updated in the Fig 2 caption (Lines 147-150).

___________________________________________________________________

Response to reviewers:

Reviewer #1: Review of PONE-D-25-09098

In this manuscript, the authors assess the historic nest use of Northern Common Eiders (Somateria mollissima borealis) on four islands in Digges Sound, which are commonly used for breeding and chick-rearing by the arctic sea ducks. Because this species has been monitored only sparsely in the past, the authors apply various paleolimnological techniques, including stable nitrogen isotopes (δ^15N), visible reflectance spectroscopy-inferred chlorophyll a (VRS-chla), and siliceous microfossils such as chrysophyte cysts, phytoliths, and protozoan plates, in addition to diatoms and metals from cores taken of nests to obtain proxies for historic nest use by eiders. The authors test whether these proxies can give reliable information on population island use by a species known to reuse nests. The chemical and organismal signatures they sampled and analyzed from the stratospheric were thorough. To further verify their chemical findings, the authors provide additional context by discussing the Little Ice Age and novel anthropogenic pressures such as increased hunting efficiency due to shotguns and motorized boats beginning around 1860, which they linked closely to fluctuations in the eider population proxies they studied. They found that by combining paleoecological data with anthropogenic and climate history, they could predict eider nest use dating back to the 13th century with the caveat that challenges with radiocarbon dating introduced multiple potential dates, highlighting the complexity of dating these archives and emphasizing the need for caution when interpreting temporal results. The authors highlight that the nest samples in their study closely align with similar findings in peat sequences in that the nests stratify vertically over time and thus provide an excellent paleoecological archive.
The authors do an excellent job justifying the necessity of developing reliable proxies to monitor populations and their chosen focal species. Their methods and statistics are sound, and they used a variety of approaches in tandem to ensure their findings could reliably link to Common Eider population presence. However, there are two main issues with the manuscript. The sample size is too small, and the interpretation of their findings along with their study question are much too broad in scope compared to what the study accomplished. With a sample size of four nests, the authors claim this study provides insight into species-wide eider nesting behavior and population dynamics. Four nests are simply insufficient to provide information on these species and population scale questions, as the patterns they found may not accurately represent the species. This is highlighted by the fact that the four nests they analyzed revealed very different histories. Far more nests would need to be sampled to find large-scale patterns. In addition, the temporal scale of their findings is limited. They dated one nest back to the 13th century, two to the 19th century, and one could only be dated to the 1980s. In the introduction, the authors justify their methods by saying we do not understand eider population dynamics pre-human presence in the Canadian sub-arctic. However, they were not able to date back to this time. Though one nest was dated to before the Industrial Revolution, they would need more nests to address the questions they present in the introduction. As the manuscript is presently, the authors leave much of the discussion as inherently speculative.
In conclusion, the presented study is an intriguing method to address a problem in assessing vitally important historical population trends that could have lasting conservation impacts. The study offers a valuable preliminary investigation into this method, and the authors could strengthen future work by expanding their sample size. The authors need to re-frame the manuscript before publication by narrowing their interpretive scope.

RESPONSE: We are thankful for the referee’s close attention to the manuscript and their valuable remarks.

**Major Comments:**

Introduction line 115– Objective (iii) – “..track long-term changes in the nest environment and populations.” is not covered in the study design or sample size. I suggest removing this goal entirely or mentioning that this was initially a goal that could not be addressed by the limited sample size.

RESPONSE: Thank you for your suggestion. Following the reviewer’s recommendation, we have modified the objectives to better reflect the nature of the study as a pilot project by rephrasing the questions and by removing the word “populations” since tracking populations may be difficult to achieve with only 4 nests (Lines 119-127).

Materials and Methods lines 162-172 – Somewhere in this paragraph an explanation for the limited sample size should be included.

RESPONSE: Yes, we completely agree. We have included an explanation for the limited sample size (lines 182-188). Nests were selected based on their proximity to a freshwater pond and the absence of eggs at the time of sampling (i.e. either the nest was not used in 2014, or the nest had been depredated). Due to the limited availability of nests meeting these requirements on the island, the sample size was restricted. Furthermore, as this was an exploratory survey and peat analyses were costly/time consuming, we wanted to limit resources used until we knew that the techniques worked. Often siliceous macrofossils are entirely absent from peats and so we were uncertain of the outcome from collecting more than 5 nests. We wanted to also limit damage to areas surrounding eider nests. We have future plans to expand the sample size from this initial pilot study.

To address the reviewer’s comment, we added these sentences:

*Nests (n=5) were selected based on their proximity to a freshwater pond and the absence of eggs at the time of sampling (i.e., either unused in 2014 or depredated). Until the effectiveness of the approach used in this pilot study could be verified, we kept the sample size small to minimize potential habitat disturbance and resource use on the limited number of nests on the island that met our selection criteria.*

Materials and Methods – The authors should justify their choice of using CONISS and broken-stick analysis in their methodology, given that PLoS ONE readers come from diverse fields and may not be familiar with these approaches. Briefly explain why these methods were selected and how they are specifically relevant to analyzing diatom assemblages in paleoecological contexts.

RESPONSE: We thank the reviewer for highlighting this point. A brief justification was added in lines 313-317 accordingly.

Radioisotopic dating (210Pb and 14C) – This section would benefit from more explicit justification and explanation to make the methods more accessible to a broader readership. Specifically, the authors should clarify why 210Pb and 14C dating were chosen and justify using the CRS model for 210Pb dating, explicitly outlining its suitability and limitations for this type of sample. Furthermore, additional details on how intervals for dating were selected, especially given the stated difficulty in interpreting specific radiocarbon dates due to multiple possible age outcomes, would improve transparency and comprehension.

RESPONSE: Thank you for pointing this out. Yes, we agree. We have included additional general information to better clarify the use of these radioisotopic dating methods (lines 202-206 and lines 221-240).

Geochemical analyses – This section would benefit from further justification and detail to enhance clarity for the broader readership. The authors should expand on why specifically Zn, Cd, and Pb were selected for analysis, particularly if their utility as proxies for seabird inputs is widely established—providing more than one citation here would strengthen this claim. Additionally, the ‘historical shotgun sales data’ mentioned requires explicit referencing: please clearly state the source of this data and the geographic region the data encompasses, describe briefly how this dataset was obtained, and clarify any procedures used to clean or prepare these data for analysis.

RESPONSE: Thank you and we agree. We have incorporated additional information in lines 325-329 and lines 329-335. We elaborated on why we focused on Zn, Cd and Pb by adding these sentences:

*For example, seabirds generally accumulate high concentrations of trace elements due to their bioaccumulation and biomagnification throughout the food web (Mallory et al., 2015). Eider ducks specifically bioaccumulate Zn, Cd and Pb as they consume mollusks and benthic crustaceans which are enriched in these elements (Szefer et al., 2006; Mallory et al., 2015; 47).*

We also clarified where the data for historical shotgun use were derived and which geographic region this covered. We modified/added these sentences:

*Temporal fluctuations in stable Pb concentrations in each profile were plotted against historical accounts of shotgun sales from the west coast of Greenland (Moshøj et al. (2011) to examine whether changes in hunting practices (i.e., use of lead shot) and industrial heavy metal contamination can be detected in the eider nest records. Historical data (Christian Vibes, Arctic Institute archives) for rifles and shotguns that were sold or traded to Greenlandic hunters were downloaded from appendix 6 of Moshøj et al. (2011) [http://www2.dmu.dk/pub/FR808_Data_CD.zip].*

Diatoms and other siliceous indicators – This section could be strengthened by clarifying several methodological choices to make the manuscript more accessible to readers unfamiliar with paleoecological techniques. Specifically, please briefly justify why samples were collected at 1-cm intervals—was this resolution chosen to capture sufficient ecological detail, and could other resolutions have offered different insights? Additionally, clarify the exact range or criteria determining how many times the rinsing procedure was repeated before reaching neutrality. The authors should also explicitly state the method used for diatom identification—morphological or genetic—and, if morphological identification was employed, reference the key literature or databases utilized. Lastly, explain why counting a minimum of 400 diatom valves per interval was selected, and briefly justify any deviations from this count in samples with fewer diatoms present.

RESPONSE: Considering that this is a pilot study, we had no prior knowledge as to the temporal resolution of the accumulated eider nest material and, therefore, we chose to use intervals of 1-cm as a starting point. Based on the results from our four nest profiles, higher-resolution sampling (e.g., 0.5 cm intervals) could be considered for future studies. However, cutting at finer than 1.0 cm resolution poses significant practical challenges. In our case, sediment sections were cut using a bread knife. While using frozen samples and a precision tool such as a mitre saw might allow for finer slicing, it remains doubtful whether 0.5 cm resolution could be achieved reliably, and there would likely be substantial risk of cross-contamination between adjacent layers. Nevertheless, it is unlikely that a higher sampling resolution would have changed our results, and 1.0 cm increments used in this pilot study was sufficient to address our exploratory research questions.

As requested, we added more precise details on diatom sample rinsing (which are standard methods used in diatom paleolimnology) before reaching the neutrality (lines 278-281). However, we do note that samples are rinsed until litmus test indicates it is no longer acidic – we word it this way because the actual number of rinses can differ among researchers and types of materials depending on the volume of acid that can be safely removed from the underlying sediment for each rinse.

We clarified that we used morphological identification of the diatoms and indicated the key taxonomic sources for our identification (lines 293-296; references 49-53).

We further clarified the reasoning for counting approximately 400 diatom valves per interval (line 287; lines 289-293). For example, we follow our PEARL diatom counting strategies (<https://www.queensu.ca/pearl/internal/Diatom%20counting%20strategies%20for%20PEARL15Jun2010.pdf>) that is generally based on Battarbee et al. (2001). For example, for the first few eider nest samples counted, we would count 500 diatom valves - and we would keep track of the number of new species encountered in every 50 valves. The results of the raw counts were then plotted to determine at what point the number of new taxa level off. For our study, this was 400. However, we find that including this level of detail in the methods is likely not needed – although we agree that this section could be elaborated upon (we also refer to Battarbee et al. 2001 and Bennett et al., 2017).

We also elaborated further on our counting approach when diatoms became too scarce to reliably estimate percent relative abundance data. In intervals where diatoms were scarce (e.g., the second interval of DS-E1-N2 and the last interval of DS-E3-N1), we strived to reach a count of 200. Counting effort became impractical when only a few valves were encountered in ~100 fields of view.

Results: Radioisotopic dating (210Pb and 14C) – For nest DS-E3-N1, the authors note they could not reliably date the bottom-most interval due to poor-quality material. They subsequently mention using the R package ‘clam’ to generate the best-fit date for a higher interval. However, the authors do not specify precisely how the model accounted for this adjustment or missing data. Please clearly describe what assumptions or methods (e.g., extrapolation or interpolation) were used within ‘clam’ to estimate the date of this interval. Clarifying this step is vital for ensuring readers understand the robustness of the generated chronology.

RESPONSE: We appreciate the reviewer’s attention to this detail. Yes, we used “clam” to interpolate the dates between the 14C date and 210Pb results. For example, establishing a chronology for the nest profiles DS-E5-N1 and DS-E3-N1 entailed combining ^210^Pb and ^14^C dating results, as the bottom intervals of these nests lie beyond the maximum range possible for obtaining reliable dates using ^210^Pb (~150 years) methods and were within the upper range of ^14^C methods. An explanation has been added to the section (lines 398-409).

Results and Discussion – The authors rely on personal communications (pers. comm.) several times in the results and discussion sections, particularly when discussing the absence of diatoms in eider guano. While personal communications can occasionally be appropriate to supplement published evidence, they are insufficient as standalone evidence to support significant interpretations or conclusions. Please either provide published studies or empirical data supporting these statements or clarify clearly why published data were not available. Minimizing dependence on personal communications for critical aspects of the interpretation would strengthen the manuscript and improve transparency.

RESPONSE: We agree. Given that there are no published data on diatoms in eider guano, we could not use a published reference. However, we agree that it is unnecessary to refer to data that the authors generated for this particular study as (pers. comm.), and have now rewritten these sentences. For example, one of the co-authors (Matthew P. Duda) examined the guano collected for this study and found not diatoms present. Our revised sentence now reads “*In addition, diatoms did not originate from eider guano as no valves were observed in guano samples collected for this study.*” We have modified other sentences in methods and results (lines 451-454 and lines 641-643) to eliminate using the term “personal communication”.

Discussion – The authors mention in the Results that "no clear ¹³⁷Cs peaks were detected." Given that ¹³⁷Cs is commonly used as an independent chronological marker (particularly around the period of nuclear fallout), the absence of detectable peaks needs explicit interpretation. In the Discussion, please explain why ¹³⁷Cs peaks were not found in these samples. For instance, could the absence reflect local conditions, methodological issues, the nature of the nest material, or limited atmospheric deposition at this site?

RESPONSE: We agree that these additions will be helpful. The eider nests are ombrotrophic (i.e. they receive inputs solely from the atmosphere) and are highly organic – much like ombrotrophic peat deposits. These conditions were found to be well-suited for 210Pb dating (as we discuss in paper) but not for validation of the dates with 137Cs. Given these ombrotrophic, organic nature of the nest material, we rather expected that 137Cs activities would not yield pronounced and clear peaks (corresponding to the 1963 height of nuclear fallout) because under these conditions, 137Cs can be affected by post-depositional migration resulting in more diffuse activity profiles (Oldfield et al. 1979; Oleksandrenko et al. 2024).  The lack of a clear 137Cs peak been observed in many paleolimnological studies (e.g. Davis et al., 1984; Klaminder et al., 2012) as Cs-137 can be highly mobile in organic material (including lake sediment), particularly in ombrotrophic peaty type material (as mentioned above) - unlike minerotrophic peatlands where the availability of mineral matter and neutral pH prevents 137Cs mobility (Oleksandrenko et al. 2024). Moreover, ¹³⁷Cs is concentrated in lakes since they are depositional basins, so it is expected lower ¹³⁷Cs in the nest because its smaller area (Appleby, 2001). Some information has been added to lines 592-600.

Discussion – On line 478 and elsewhere, the authors discuss nutrient and moisture availability as potentially insufficient for biological assemblages during the Little Ice Age based primarily on limited data from two nests. However, they omit discussion of the fact that nest DS-E5-N1 was established well before this period, indicating at least localized continuous nesting and sufficient nutrients and moisture for biological proxies to persist throughout the Little Ice Age. Given the small sample size (four nests) and conflicting evidence from DS-E5-N1, this interpretation about nutrient availability during the Little Ice Age should be clearly marked as speculative. It would be more appropriate to explicitly acknowledge this limitation, clarifying that a larger sample size would be necessary to confidently address eider occupancy and nesting conditions during this period.

RESPONSE: Yes, we agree that we cannot make strong conclusions based on our small sample size in this pilot study. We have clarified the text of the discussion about moisture, nutrients etc. during the LIA and we more explicitly acknowledge the limitations of a small sample size of nests. However, we do not interpret differences in our nest profiles as *conflicting* evidence about nutrient availability. Rather, nutrient availability would be expected to differ between nests that were preferentially selected by eiders and nests that would be less desirable (i.e. in a more open setting and with less nesting material). During the LIA, conditions would have been harsher, so perhaps populations would be lower and fewer less-desirable nests were occupied. Given that nutrients and moisture for diatom, chrysophyte, and protozoa growth are supplied by the nesting eiders, it may be conjectured that nests with no biological proxies present (esp. during the LIA) was not preferentially selected during this relatively severe climate period. Some information has been added to lines 672-678 and 683-694.

Overall manuscript – The sentence at line 473 in the Discussion very clearly and effectively articulates the core rationale and intended scope of the study. I recommend the authors consistently emphasize and reiterate this perspective throughout the manuscript—particularly in the Introduction, Results, and Conclusions sections—to align their stated goals with the scope of their findings. Reinforcing this point more explicitly throughout the manuscript will further clarify the study’s contributions and limitations.

RESPONSE: Thank you once again for your very thorough and thoughtful comments. As suggested, we have changed the text in strategic parts of the manuscript to reinforce that this is a pilot project and that eider nests can be effective archives for palaeoecological proxies.

**Figures** – The quality of several figures, particularly Figure 8, needs improvement to enhance readability and accessibility. Currently, the resolution of all figures is low, making text and symbols difficult to discern. In addition, the color choices in Figure 8 are challenging to distinguish, especially for readers with color vision deficiencies. Consider using a colorblind-friendly color palette.

RESPONSE: Thank you very much for your suggestion. Figures have been remade to enhance their readability and resolution. In all the figures, including figure 8, the viridis palette has been used for accessibility to colorblind people.

Figure 9 – This figure currently displays a timeline spanning over 800 years, making it very challenging to interpret trends occurring around the introduction of regulated hunting due to compressed visual resolution. To enhance clarity and emphasize patterns most relevant to their hypotheses about regulated hunting impacts, the authors should consider dividing this figure into multiple panels and/or providing an inset zoomed specifically around the post-industrial era and following the introduction of regulated hunting. This visual restructuring would significantly improve interpretability, highlighting key temporal changes related to anthropogenic impacts.

RESPONSE: We appreciate your suggestion. In accordance with your recommendations, Figure 9 has been revised to include two additional zoomed-in panels that highlight post-industrial era trends.

**Minor Comments:**

Line 55 – The authors introduce the economic and cultural significance here, yet they do not explain this significance until line 68. Remove this line or move it to line 68.
RESPONSE: Thank you for your comment. We carefully reviewed lines 55 to 68 and could not identify a sentence introducing the economic or cultural significance of eiders prior to line 68. The discussion of their importance to Indigenous communities begins around line 71, and we believe the subsequent transition into hunting pressure flows logically. If there is a specific sentence you were referring to, we would be happy to revisit it in a future revision.

Line 85 – The word ‘local’ is stated twice. I suggest removing one.
RESPONSE: Thanks for your recommendation, the repeated word has been removed.

Digges Sound: site description and brief history of the region lines 129-132 – Mention specifically any help received by locals, especially the Ivujivik community.
RESPONSE: Help accessing sites was received from community members in Ivujivik, this has been added in line 136. Community member names are listed in the acknowledgements.

Figure 2 – Add provincial and state borders to further clarify geographic location.

RESPONSE: Thank you very much for your suggestion. In the new version of the map, provincial and state borders are included.

Lines 142-145 – Cite nest selection behavioral

RESPONSE: We appreciate your recommendation and have included referenced information accordingly about nest selection behavioral to lines 153-170.

Lines 142-145 – Add a sentence about the frequency of new bowl selection by eider females (i.e., how often to females start an entirely new nest?); cite.

RESPONSE: Thank you for your suggestion. We don’t have specific statistics on this because it is so rare to observe. Most eiders select established, pre-existing nest cups as Fast et al. (2010) pointed out in the paper that we cited.

Materials and Methods lines 152-172 – I suggest specifying when eider breeding occurs and whether or not data collection overlapped with the breeding season.
RESPONSE: Thank you very much for your suggestion. We have added a comment about the sampling overlapping with the end of the active breeding season (lines 189-190).

Materials and Methods line 162 – Add sample size (e.g. n = 4) and briefly mention one nest was not used.

RESPONSE: Thank you for the suggestion. We have included the sample size in line 182. We mention that DS-E1-N1 was not analyzed in line 380-383.

Line 172 – Justify 4°C

RESPONSE: The general recommendation is to store sediment in the dark at this temperature. Reference has been added in line 199.

Chlorophyll a line 249 – Specify which variables.

RESPONSE: Thank you for pointing out this out and we have now clarified this sentence (lines 340-343).

Stable nitrogen isotopes line 266 – It would be beneficial for the authors to briefly clarify the meaning of "AIR" as the reference standard for δ¹⁵N measurements, specifying explicitly that it refers to atmospheric nitrogen.
RESPONSE: Agreed and we have added the specification (line 359).

Lines 268-271 – ..”to facilitate comparisons..” is used two sentences in a row. I suggest rephrasing one of the two sentences.

RESPONSE: Thank you very much for your suggestion. We have modified the sentence.

Results Line 298 – The authors should briefly explain or define "CE" (Common Era) upon first mention in the manuscript. A simple parenthetical clarification such as "(CE, Common Era)" would enhance readability.

RESPONSE: We appreciate the insight. Accordingly, we have added this clarification.

Results Line 298 – Define “cal BP” upon first use.
RESPONSE: We appreciate the insight. Accordingly, we have added this definition in line 397.

Results line 368 – Justify use of CONISS and broken stick analysis; briefly explain these methods in the context of the field.
RESPONSE: Following your suggestion, we have included a brief explanation in lines 475 and 477.

Discussion line 478 – Briefly define "humify" or "humification" to improve readability for non-specialists unfamiliar with soil and peat-forming processes.

RESPONSE: We appreciate your suggestion. We have included it in lines 589-590.

Discussion line 487 – CRS is already defined in the materials and methods. I recommend writing the complete phrase ‘constant rate of supply model’ each time, as it is infrequently used. Alternatively, use CRS here without definition since it has already been defined.
RESPONSE: Thank you for your insight. It has been incorporated in line 220.

Discussion line 500 – Clarify which specific result detected the variation in the duration of nesting occupancy.
RESPONSE: We thank the reviewer for the observation. As stated throughout the manuscript, all the proxies used to detect variation in nesting occupancy duration (i.e. diatoms, δ¹⁵N, chlorophyll-a…) are those already described in the Methods and Results sections.

Discussion lines 571-574 – This sentence is especially clear and effectively contextualizes the results within the broader literature. This type of explicit connection to past studies greatly enhances the reader’s understanding and interpretation of the findings and adds valuable additional context to the observed results in this manuscript.
RESPONSE: We really appreciate the referee’s careful reading and insight.

Discussion lines 576-577 – The authors do not have enough data to claim that they, “reconstruct[ed] long-term trends in this species’ nesting behavior and ecological influence.” I strongly suggest rephrasing or removing.
RESPONSE: Thank you for your valuable insight. We have rephrased the sentence (Line 713).

Discussion line 580 – Spell out “Little Ice Age.”
RESPONSE: Thank you for your comment. We have spelled out Little Ice Age in line 718.

Discussion line 634 – I suggest substituting the use of the informal ‘and/or’ for “may be a result of either the ingestion of spent Pb..or from eiders carrying embedded Pb shot.”
RESPONSE: We thank the referee for the insight. Following her recommendation, the sentence has been changed (lines 771-773).

Discussion line 642 – A period is missing.

RESPONSE: We appreciate the comment. The missing period has been included.

References – Address inconsistencies in journal name abbreviations and article title capitalization schemes. For example, Conservation Biology is abbreviated in reference 5 but not in reference 3. Some article titles are sentence case while others are in title case.

RESPONSE: Thank you very much for your valuable comment. References have been reviewed and corrected.

**Reviewer #2: PONE-D-25-09098**

The authors used paleoecological techniques to investigate historical usage indicators of Common Eider nests which can provide insight into historical ecological conditions, frequency of use, age, and population sizes. This pilot study is novel and innovative and deserves to be added to the body of literature. Potential applications for conservation and management of this culturally and ecologically valuable species are promising. I have a single minor suggestion, and found 2 typos. Overall, well-written and easy to follow.
RESPONSE: We are grateful for the referee’s thoughtful assessment and constructive feedback.

General comment: It would be nice to have a short section explaining what the different groups/indicators/taxa signify ecologically and have a quick reminder at the beginning of the discussion for readers (like myself) who are unfamiliar with diatoms and siliceous indicators.

RESPONSE: We thank the reviewer for highlighting this point. We have included information about diatoms and the other siliceous indicators in lines 252-269 and lines 627-631.

Line 342: unnecessary period following genus name (typo).

RESPONSE: We acknowledge the reviewer’s remark and have addressed it.

Line 642: Missing period at end of sentence

RESPONSE: We appreciate the referee for pointing that out. The missing period has been included.
